# Exogenous DA-6 Improves the Low Night Temperature Tolerance of Tomato Through Regulating Cytokinin

**DOI:** 10.3389/fpls.2020.599111

**Published:** 2021-02-04

**Authors:** Jiazhi Lu, Pengxiao Guan, Jiamao Gu, Xiaolong Yang, Feng Wang, Mingfang Qi, Tianlai Li, Yufeng Liu

**Affiliations:** ^1^College of Horticulture, Shenyang Agricultural University, Shenyang, China; ^2^Key Laboratory of Protected Horticulture of Education Ministry and Liaoning Province, Shenyang, China; ^3^Collaborative Innovation Center of Protected Vegetable Surrounds Bohai Gulf Region, Shenyang, China

**Keywords:** DA-6, tomato, low night temperature, hormone, stress tolerance

## Abstract

Low night temperature (LNT) causes environmental stress and has a severe and negative impact on plant growth and productivity. Synthetic elicitors can regulate plant growth and induce defense mechanisms from this type of stress. Here, we evaluated the effect of the exogenous growth regulator diethyl aminoethyl hexanoate (DA-6) in tomato leaf response to LNT stress. Our results showed that exogenous DA-6 activates the expression of chlorophyll synthesis and photosystem-related genes, and results in higher photosynthetic activity and chlorophyll production. Furthermore, DA-6 can regulate the synthesis of endogenous cytokinin (CTK) and the expression of decomposition genes to stabilize chloroplast structure, reduce oxidative damage, and maintain the photochemical activity of tomato leaves under LNT stress. DA-6 maintains a high level of ABA content and induces the expression of *CBF* genes, indicating that DA-6 may participate in the cold response signaling pathway and induce the expression of downstream low temperature response genes and accumulation of compatible osmolytes. This study unravels a mode of action by which plant growth regulators can improve low temperature tolerance and provides important considerations for their application to alleviate the harmful effects of cold stress.

## Introduction

Low temperature is a major environmental stress that severely decreases plant growth and productivity. It is also a determining factor in the geographical distribution of plants. Under low temperature conditions, plants exhibit a variety of cold-induced physiological and biochemical responses, such as production of reactive oxygen species (ROS) and changes in osmolytes (Browse and Xin, 2001). This type of stress can disrupt main photosynthetic processes such as thylakoid electron transport, the Calvin cycle, and stomatal conductance (Kratsch and Wise, 2000; Allen and Ort, 2001; Lu et al., 2020b).

To survive cold stress, plants trigger a series of complex physiological and biochemical responses (Zhu, 2016). Some of these responses involve changes in gene expression and posttranscriptional processes that are abscisic acid (ABA)-dependent (Dreyer and Dietz, 2018). ABA activates the expression of stress response genes mainly through pyrabactin resistance1/pyr1-like/regulatory components of ABA receptors (PYR/PYL/RCAR) and clade A protein phosphatases 2C (PP2C) (Wang et al., 2018). C-repeat-binding factors (CBF/DREB) are ABA independent and can also induce the expression of genes related to stress tolerance (Kim, 2007; Wisniewski et al., 2011). The cold stress-induced CBF transcription factors directly activate the expression of downstream cold-regulated (COR) genes. Knocking out all three CBF genes leads to an extreme sensitivity to cold stress (Zhu, 2016; Zhao et al., 2016), while overexpressing CBF genes leads to an enhanced cold tolerance (Jaglo-Ottosen et al., 1998; Gilmour et al., 2000). CBF3 improves cold tolerance in tomato by regulating COR genes and binding to the DNA regulatory element known as CRT/DRE containing the same motif (CCGAC) (Xiong and Fei, 2006). Additionally, growth-related phytohormones, such as gibberellins, brassinosteroids, auxins, and cytokinins (CTK) are also involved in defense responses against cold stress (Eremina et al., 2016).

Several studies have shown the positive effects of applying exogenous regulators, e.g., brassinolide (BR), ABA, calcium, and NO (Uchida et al., 2002; Larkindale and Huang, 2005; Liu et al., 2015; Siddiqui et al., 2018) to reduce damage from abiotic stress in plants (Kumar et al., 2011). Diethyl aminoethyl hexanoate (DA-6) is a plant growth regulator that has been applied to a wide range of agricultural crops. It promotes cell growth and division as well as protein and nucleic acid synthesis (Zhang et al., 2008; Jiang et al., 2012). DA-6 also promotes germination and seedling establishment in aged soybean seeds by enhancing the hydrolysis of triacylglycerol and the conversion of fatty acids to sugars (Zhou et al., 2019). In combination with GA3, DA-6 has been shown to alleviate the adverse effect of EDTA on plant growth (He et al., 2013). Applied with 6-BA, it can reduce cadmium toxicity by retaining it within the cell wall (Li et al., 2018). The effectiveness of DA-6 to promote photosynthetic activity has been well shown (Jiang et al., 2012). However, its efficiency and positive effect in plant response to low temperature stress has been poorly studied.

Tomato (*Solanum lycopersicum*) is an important crop and widely consumed fruit. Its genetics, physiology, and biochemistry have been well studied (Colombie et al., 2015). Tomato plants show low tolerance to cold stress (Shah et al., 2015). The common low night temperature (LNT) phenomenon in greenhouse vegetable cultivation during the winter and spring in northern China has significantly reduced fruit yield. In this study, we treated tomato seedlings with DA-6 at LNT stress to assess its effect in tomato response to cold stress. We evaluated the following parameters: chloroplast structure, chlorophyll (Chl) and hormone levels, and gene expression related to low-temperature response.

## Materials and Methods

### Plants and Growth Conditions

Seeds of tomato “cv. Liaoyuanduoli,” a popular variety in Northeast China, were germinated and grown in pots (mixture of three parts peat to one part vermiculite, receiving Hoagland’s nutrient solution) under cool-white fluorescent light (600 μmol m^–2^ s^–1^, 12 h light/12 h dark) at 28°C/18°C and 60% relative humidity in a growth chamber (KuLan Beijing). Tomato seedlings at the four-leaf stage were separated into three portions, with 20 pots each. The first and second groups were sprayed with an equivalent volume of distilled water. The third group was sprayed with 10 mg/L of DA-6; sprayed twice a day for 3 days (6:00 am and 18:00 pm). After exogenous application, the first group of plants was cultivated under normal conditions (28°C/18°C). The second and third groups were subjected to LNT treatment at 6°C. The treatment was performed 12 h a day (from 18:00 pm to 06:00 am) for 12 days.

Throughout the experiment, all measurements were performed on the fourth fully expanded functional leaves using five replicates from different pots. Leaf samples at 6:00 am on days 0, 3, 6, 9, and 12 during the treatment were used freshly or immediately frozen in liquid nitrogen and stored at −80°C. Five biologically independent replicates for each treatment were collected.

### Measurement of Chlorophyll Content

The chlorophyll content of the plant leaves was measured after treatment for 12 days. Take the fourth functional leaves, and immerse it in acetone and anhydrous ethanol mixture (1:1) and put in the dark until leaves turned completely white. The absorbance of the supernatant was measured at 440.5, 663, and 645 nm and recorded as OD_440.5_, OD_663_, and OD_645_, respectively (Fadeel, 1962; Porra, 2002).

### Measurement of Gas Exchange Parameters

The net photosynthetic rate (Pn), transpiration rate (Tr), stomatal conductance (Gs), and intercellular CO_2_ concentration (Ci) were measured using GFS-3000 and DUAL-PAM-100 synchronous measuring instrument (Heinz Walz, Effeltrich, Germany) with constant irradiation (228 μmol photons m^–2^ s^–1^, PAR) *in vivo*. Leaf temperature and CO_2_ concentration were maintained at 28°C and 500 ppm (Yang et al., 2020).

### Measurement of Chlorophyll Fluorescence and P700 Parameters

Chlorophyll fluorescence and P700 redox state of leaves were measured using the Dual-PAM-100 (Heinz Walz, Effeltrich, Germany) as described by Yamori et al. (2015), with small-scale modifications. The maximum quantum yield of PSII (Fv/Fm), effective photochemical quantum yield of PSII [Y(II)], and coefficient of photochemical fluorescence quenching (qP) were determined with the MAXI-Imaging-PAM (blue LED version) and the imaging fluorometer software Win (Heinz Walz, Effeltrich, Germany) as previously described (Lu et al., 2020a). After leaves were dark adapted for 30 min, a saturating pulse (10,000 μmol photons m^–2^ s^–1^, 300 ms) was applied to obtain maximal fluorescence and maximal P700 changes. The actinic light (AL) for measurements of chlorophyll fluorescence was 228 μmol photons m^–2^ s^–1^ (635 nm). The dark-adapted and light-adapted maximal fluorescence (Fm and Fm′) were obtained with saturating pulse. The dark-adapted and light-adapted initial fluorescences (Fo and Fo′) were measured by switching on the modulated irradiation of less than 0.1 μmol⋅m^–2^⋅s^–1^PPFD on the leaf surface. Pm and Pm′ are analogous to Fm and Fm′, respectively, and they were given by the same means as the former fluorescence parameters by applying a saturation pulse after pre-illumination with far-red light (Lu et al., 2017).

The chlorophyll fluorescence parameters were calculated as follows: Fv/Fm = (Fm − Fo)/Fm, Y(II) = (Fm − Fs)/Fm′, NPQ = (Fm − Fm′)/Fm′, qP = (Fm′ − Fs)/(Fm′ − Fo′), Y(I) = (Pm′ − P)/Pm, Y(ND) = P/Pm, Y(NA) = (Pm − Pm′)/Pm, ETR(I) = Y(I) × PAR × 0.84 × 0.5, ETR(II) = Y(II) × PAR × 0.84 × 0.5.

### Measurement of Chloroplast Ultrastructure

The fourth fully expanded leaves from the top of the plants were randomly selected for electron microscopic examination on the 12th day of treatment. The leaf samples were sectioned, and then the samples were observed in transmission electron microscopy (Model H7650; Hitachi; Japan) at 75 kV according to the method described by Wang F. et al. (2020).

### Analysis of Superoxide Anion (O_2_^–^) and Hydrogen Peroxide (H_2_O_2_)

The accumulation of superoxide (O_2_^–^) and hydrogen peroxide (H_2_O_2_) in the fresh leaves was detected using nitroblue tetrazolium (NBT) and 3,30-diaminobenzidine (DAB) staining, respectively, as previously described (Mostofa et al., 2015).

H_2_O_2_ content was determined spectrophotometrically after potassium iodide treatment following previously published protocols (Ibrahim and Jaafar, 2012). Briefly, the fresh leaf tissue was ground in 0.1% trichloroacetic acid, and the homogenate was centrifuged at 15,000 *g* for 15 min at 4°C, and the supernatant was used to measure H_2_O_2_ levels. The generation rate of O_2_^–^ was determined according to Elstner and Heupel (1976) by monitoring the nitrite formation from hydroxylamine in the presence of O_2_^–^.

### Measurement of Hormone Contents

The contents of different phytohormones were analysis by ELISA as previously described (Yang et al., 2001). The mouse monoclonal antigens and antibodies against IPA, ZR, JA, BR, IAA, GAs, and ABA, and IgG-horseradish peroxidase used in ELISA were produced at the Phytohormones Research Institute (China Agricultural University). The results are the means of five replicates.

### Total RNA Extraction and Real-Time Polymerase Chain Reaction (qRT-PCR) Analysis

Total RNA was extracted from tomato leaves using an RNAprep Pure Plant Kit (Tiangen Biotech) following the manufacturer’s recommendations. The extracted RNA was reverse-transcribed using a PrimeScript^TM^RT reagent kit with gDNA Eraser (TaKaRa). qRT-PCR experiments were performed on an Applied Biosystems 7500 Real-Time PCR System with an SYBR Green PCR Master Mix Kit (TaKaRa). Actin, a housekeeping gene, was used to normalize changes in expression. Each pair of primer was designed using Primer Express 5.0 (Applied Biosystems, United States). The primer sequences are listed in Supplementary Table 1.

### Data Analysis and Graphics

The quantitative assessment was conducted on randomly selected samples; values are the mean ± SD of five replicates. The data were analyzed using SPSS 20 Software (IBM SPSS STATISTICS, United States) by ANOVA; statistically significant difference was set at a probability level of 0.05. The figures were drawn with Origin 9.0 Software (Origin Lab, Northampton, MA, United States).

## Results

### Effects of DA-6 on Gas Exchange

**FIGURE 1:**
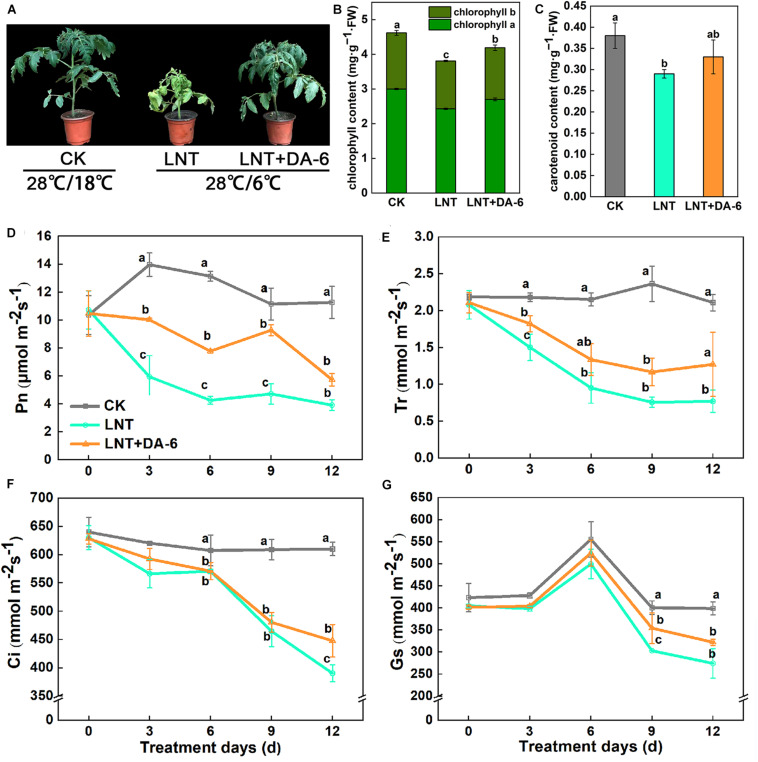
Effect of diethyl aminoethyl hexanoate (DA-6) treatment on chlorophyll and photosynthesis in tomato leaves under low night temperature. **(A)** Plant phenotype. **(B)** Chlorophyll content. **(C)** Carotenoid content. **(D)** Net photosynthetic rate (Pn). **(E)** Transpiration rate (Tr). **(F)** Intercellular CO_2_ concentration (Ci). **(G)** Stomatal conductance (Gs). Data are the means of five replicates at 12 days with standard errors shown by vertical bars. Differences among treatments were analyzed by the one-way ANOVA comparison test (*P* < 0.05). Different letters indicate significant differences among treatments. CK, the plant grown at optimal temperature (28°C/18°C); LHT, the plant grown at low night temperature (28°C/6°C). LHT + DA-6, the plant pretreated with DA-6 and grown at LHT.

**FIGURE 2:**
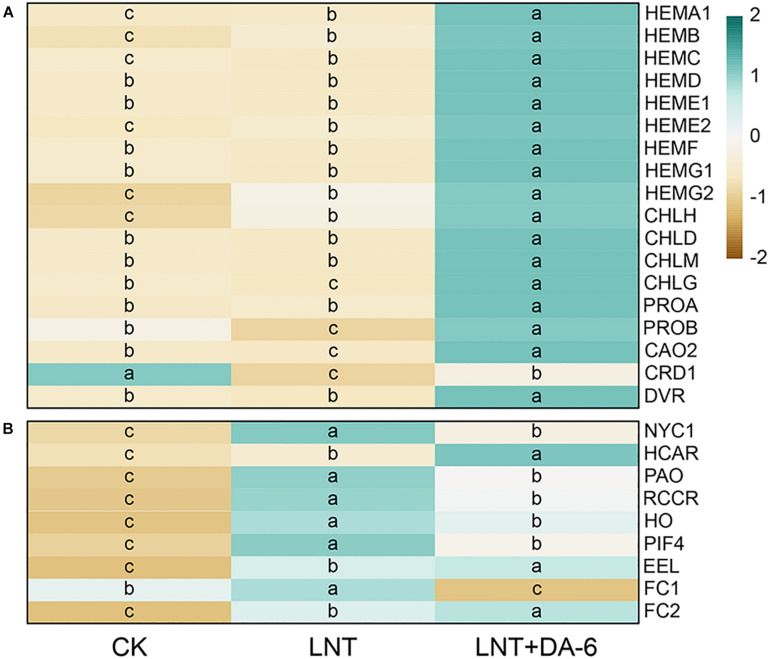
Effect of DA-6 treatment on related gene expression of chlorophyll in tomato leaves under low night temperature. **(A)** Chlorophyll synthesis-related genes. **(B)** Chlorophyll decomposition-related genes. Data are the means of five replicates at 12 days. Differences among treatments were analyzed by the one-way ANOVA comparison test (*P* < 0.05). Different letters indicate significant differences among treatments. CK, the plant grown at optimal temperature (28°C/18°C); LHT, the plant grown at low night temperature (28°C/6°C); LHT + DA-6, the plant pretreated with DA-6 and grown at LHT.

Plant growth with the LNT + DA-6 treatment is better than the LNT treatment (Figure 1A). The total levels of Chl, Chl a, carotenoid, and Chl a/b in leaves after LNT+DA-6 treatment were significantly higher than those with LNT treatment (Figures 1B,C). Photosynthetic rate (Pn), stomatal conductance (Gs), intercellular CO_2_ concentration (Ci), and transpiration ratio (Tr) were significantly lower under LNT conditions compared to the control. However, the application of exogenous DA-6 minimized the negative effect in these parameters (Figures 1D–G). All chlorophyll synthase-encoding genes except *CHLH*, *HEMB*, *HEME2*, and *HEMG2* were remarkably downregulated with the LNT treatment. The expression level of chlorophyll synthesis gene in LNT + DA-6 treatment was higher than other treatments. Under LNT conditions, the expression levels of the chlorophyll-degrading genes *NYC1*, *HCAR*, *PAO*, *RCCR*, *HO*, *PIF4*, *NAO16*, *EEL*, *FC1*, and *FC2* were higher than that of the control. Compared with the LNT treatment, plants treated with LNT + DA-6 showed reduced expression of these same genes (Figure 2).

### Effects of DA-6 on Photosystem Activity

**FIGURE 3:**
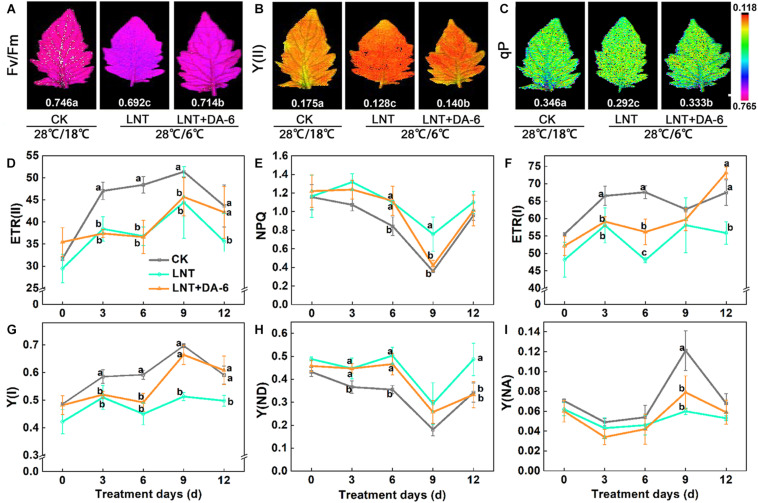
Effect of DA-6 treatment on the photosystem activity in tomato leaves under low night temperature. **(A)** Maximum photochemical efficiency of PSII (Fv/Fm). **(B)** Effective quantum yield of PSII photochemistry [Y(II)]. **(C)** Coefficient of photochemical fluorescence quenching (qP). Data are measured on the 12th day. **(D)** The electron flow through PSII [ETR(II)]. **(E)** Non-photochemical quenching (NPQ). **(F)** The electron flow through PSI [ETR(I)]. **(G)** Quantum yield of PSI photochemistry [Y(I)]. **(H)** The quantum yield of PSI non-photochemical energy dissipation due to donor-side limitation [Y(ND)]. **(I)** The quantum yield of PSI non-photochemical energy due to acceptor-side limitation [Y(NA)]. Data are the means of five replicates with standard errors shown by vertical bars. Differences among treatments were analyzed by the one-way ANOVA comparison test (*P* < 0.05). Different letters indicate significant differences among treatments. CK, the plant grown at optimal temperature (28°C/18°C); LHT, the plant grown at low night temperature (28°C/6°C); LHT + DA-6, the plant pretreated with DA-6 and grown at LHT.

Photochemical efficiency (Fv/Fm), photosystem I (PSI), and photosystem II (PSII) effective quantum yield, photochemical quenching (qP), and electron transport rate (ETR) in both PS I and II were lower under LNT conditions. Conversely, non-photochemical fluorescence quenching (NPQ) was higher at LNT. All of these parameters were higher when exogenous DA-6 was applied (Figures 3A–G). Likewise, LNT treatments increased the quantum yield of PSI non-photochemical energy dissipation due to donor-side limitation [Y(ND)] and reduced the quantum yield of PSI non-photochemical energy dissipation due to acceptor-side limitation [Y(NA)] (Figure 3H). However, such changes in Y(ND) and Y(NA) were reversed in plants treated with LNT + DA-6 (Figure 3I). These results indicate that exogenous DA-6 alleviates the photodamage produced by LNT to the PSI donor side.

**FIGURE 4:**
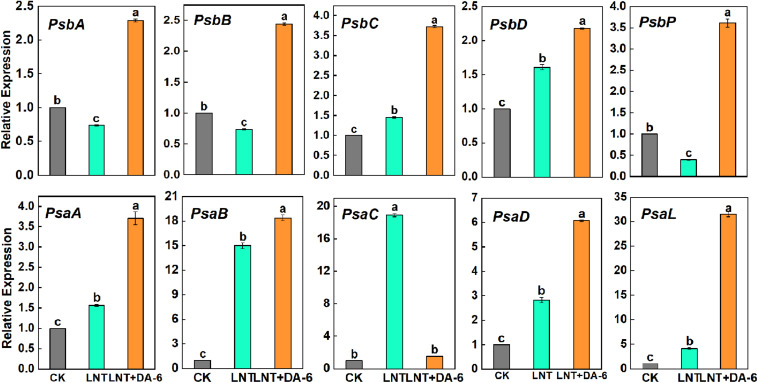
Effect of DA-6 treatment on related gene expression of photosystem in tomato leaves under low night temperature. *PsbA*, *PsbB*, *PsbC*, *PsbD*, and *PsbP*: photosystem II-related gene. *PsaA*, *PsaB*, *PsaC*, *PsaD*, and *PsaL*: photosystem I-related gene. Data are the means of five replicates at 12 days with standard errors shown by vertical bars. Differences among treatments were analyzed by the one-way ANOVA comparison test (*P* < 0.05). Different letters indicate significant differences among treatments. CK, the plant grown at optimal temperature (28°C/18°C); LHT, the plant grown at low night temperature (28°C/6°C); LHT + DA-6, the plant pretreated with DA-6 and grown at LHT.

Gene expression levels of photosystem-related genes suggest that LNT stress enhanced the expression of *psbC*, *psbD*, *psaA*, *psaB*, *psaC*, *psaD*, and *psaL*, and downregulated the expression of *psbA*, *psbB*, and *psbP*. With the exception of *psbC*, all photosystem-related genes showed higher expression levels under the LNT + DA-6 treatment (Figure 4).

### Effects of DA-6 on Chloroplast Ultrastructure

**FIGURE 5:**
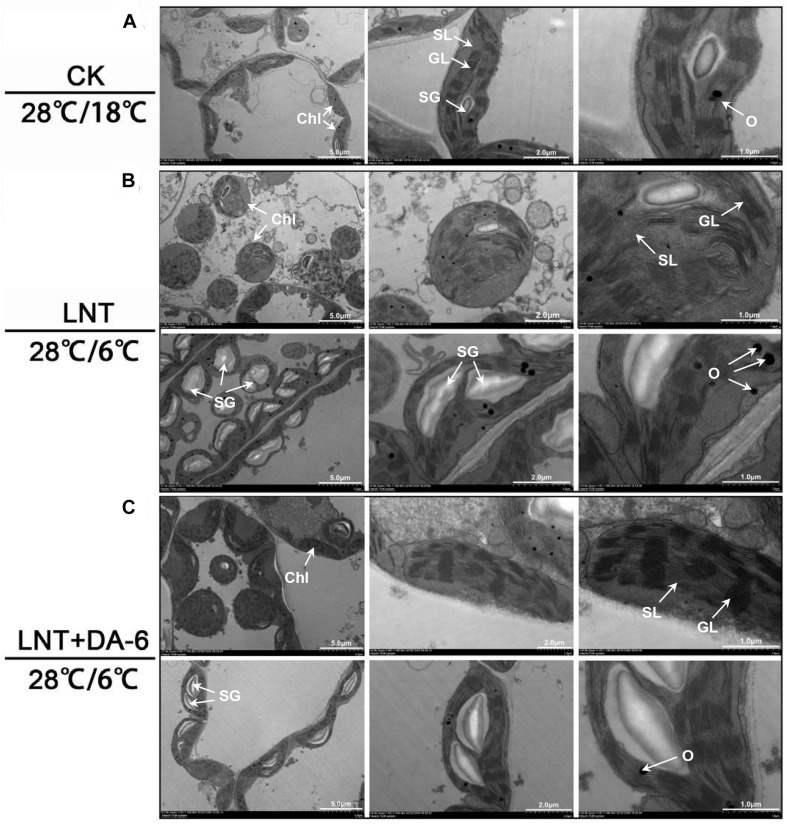
Effect of DA-6 treatment on chloroplast ultrastructure in tomato leaves under low night temperature. **(A)** The chloroplast structures in tomato leaves under 28°C/18°C. **(B)** The chloroplast structures in tomato leaves under 28°C/6°C. **(C)** The chloroplast structures in tomato leaves treated with DA-6 at 28°C/6°C. Chl, chloroplast; SL, stroma lamella; GL, grana lamellae; SG, starch grain; O, osmiophilic granules.

Transmission electron microscopy (TEM) images showed that chloroplasts in seedlings grown in normal conditions were slender, had smoothly arranged grana and stroma lamellae, and a lower number of starch grains and osmiophilic granules (Figure 5A). Under LNT treatment, chloroplasts looked swollen and round. Chloroplast width, starch granule size, and stack width increased significantly, and the accumulation of osmiophilic granules was high. Thylakoids were relaxed, and membranes were degraded (Figure 5B and Table 1). Plants treated with LNT + DA-6 exhibited elliptical and structurally stable chloroplasts. Chloroplast width, starch granule size, stack width, and the number of osmiophilic granules were lower (Figure 5C and Table 1).

**TABLE 1:** Effect of DA-6 treatment on ultrastructure of chloroplast of tomato leaves.

	Chloroplast							
	
Treatment	Length (μM)	Widen (μM)	Length/Widen L/W	Area (μM^2^)	size (μM^2^)	Starch grains per cell	Starch grains/	Number of osmiophilic granules
CK	5.77 ± 0.4^*a*^	2.02 ± 0.1^*c*^	2.87 ± 0.3^*a*^	16.21 ± 0.2^*a*^	1.65 ± 0.1^*b*^	10.67 ± 0.9^*b*^	0.49 ± 0.04^*b*^	4.67 ± 0.4^*c*^
LNT	4.84 ± 0.5^*a*^	5.11 ± 0.5^*a*^	1.22 ± 0.2^*c*^	15.86 ± 0.3^*a*^	5.41 ± 0.6^*a*^	20.67 ± 1.7^*a*^	0.87 ± 0.03^*a*^	17.33 ± 0.9^*a*^
LNT + DA-6	5.88 ± 0.2^*a*^	2.94 ± 0.1^*b*^	2.00 + 0.1^*b*^	15.22 ± 0.8^*a*^	2.50 ± 0.2^*b*^	11.00 ± 0.8^*b*^	0.57 ± 0.04^*b*^	6.33 ± 0.5^*b*^

### Effects of DA-6 on Active Oxygen Accumulation

**FIGURE 6:**
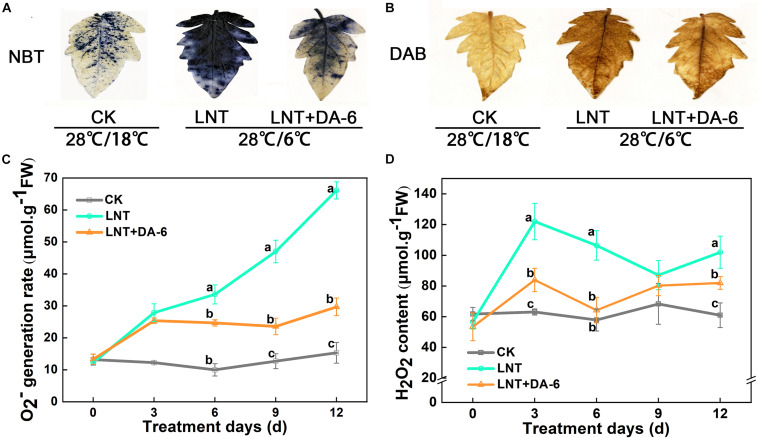
Effect of DA-6 treatment on active oxygen accumulation in tomato leaves under low night temperature. **(A)** Histochemical staining of tomato leaves under various treatment conditions with tetranitroblue tetrazolium chloride (NBT). **(B)** Histochemical staining of tomato leaves under various treatment conditions with diaminobenzidine (DAB). **(C)** O_2_^–^ generation rate in leaves. **(D)** H_2_O_2_ content in leaves. Data are the means of five replicates with standard errors shown by vertical bars. Differences among treatments were analyzed by the one-way ANOVA comparison test (*P* < 0.05). Different letters indicate significant differences among treatments. CK, the plant grown at optimal temperature (28°C/18°C); LHT, the plant grown at low night temperature (28°C/6°C); LHT + DA-6, the plant pretreated with DA-6 and grown at LHT.

Photosystem inhibition leads to the accumulation of excess electrons, which combine with oxygen molecules to produce ROS (Foyer et al., 2002). Histochemical staining with tetranitroblue tetrazolium chloride (NBT) and diaminobenzidine (DAB) revealed increased levels of O_2_^–^ and H_2_O_2_ in LNT-treated plants (Figures 6A,B). Similarly, measurements of O_2_^–^ and H_2_O_2_ also showed that the O_2_^–^ generation rate and H_2_O_2_ content in leaves were significantly higher under LNT treatment, whereas LNT + DA-6 treatment lowered the O_2_^–^ generation rate and H_2_O_2_ content (Figures 6C,D).

### Effects of DA-6 on Hormonal Content

**FIGURE 7:**
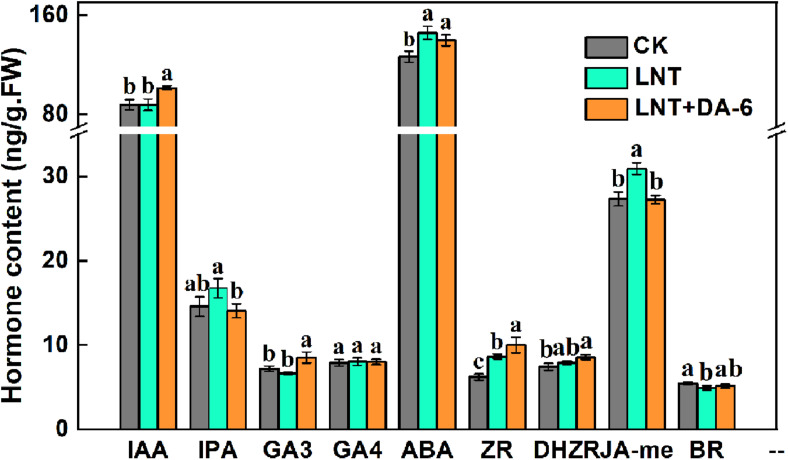
Effect of DA-6 treatment on hormone contents in tomato leaves under low night temperature. Data are the means of five replicates at 12 days with standard errors shown by vertical bars. Differences among treatments were analyzed by the one-way ANOVA comparison test (*P* < 0.05). Different letters indicate significant differences among treatments. CK, the plant grown at optimal temperature (28°C/18°C); LHT, the plant grown at low night temperature (28°C/6°C); LHT + DA-6, the plant pretreated with DA-6 and grown at LHT.

Plants under LNT had a higher amount of ABA, zeatin riboside (ZR), and jasmonate (JA), and low BR content. LNT + DA-6 treatment increased ZR and lowered JA and IPA concentrations. Indole-acetic acid (IAA), gibberellin (GA3), and dihydrozeatin riboside (DHZR) content showed no significant change under the LNT treatment, but significantly increased with the LNT + DA-6 treatment (Figure 7).

### Effect of DA-6 on Gene Expression

**FIGURE 8:**
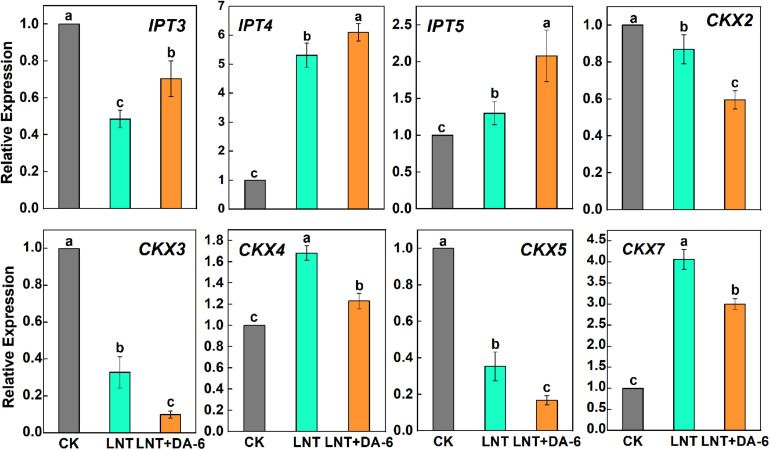
Effect of DA-6 treatment on related gene expression of CTK synthesis and decomposition in tomato leaves under low night temperature. *IPT3*, *IPT4*, and *IPT5*: CTK synthesis pathway gene. *CKX2*, *CKX3*, *CKX4*, *CKX5*, and *CKX7*: CTK decomposition pathway gene. Data are the means of five replicates at 12 days with standard errors shown by vertical bars. Differences among treatments were analyzed by the one-way ANOVA comparison test (*P* < 0.05). Different letters indicate significant differences among treatments. CK, the plant grown at optimal temperature (28°C/18°C); LHT, the plant grown at low night temperature (28°C/6°C); LHT + DA-6, the plant pretreated with DA-6 and grown at LHT.

Analysis of plant hormonal levels showed that ZR content changed greatly with each treatment, so we further measured the expression of CTK synthesis and decomposition pathway-related genes. Expression of IPT3, CKX2, CKX3, and CKX5 genes decreased at LNT. On the other hand, the expression of IPT4, IPT5, CKX4, and CKX7 genes was higher at LNT (Figure 8). Tomato leaves treated with exogenous DA-6 under LNT showed an increase in the CTK content and resulted in significant upregulation of all CTK synthesis genes and significant downregulation of decomposition genes (Figure 8).

**FIGURE 9:**
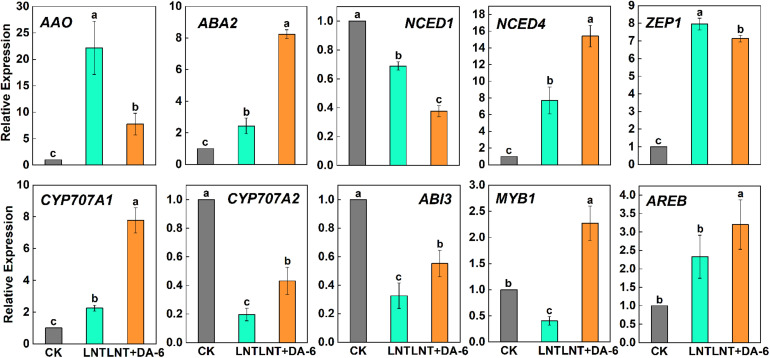
Effect of DA-6 treatment on related gene expression of ABA in tomato leaves under low night temperature. *AAO*, *ABA2*, *NCED1*, *NCED4*, *ZEP1*: ABA synthesis pathway gene. *CYP707A1*, *CYP707A2*: ABA decomposition pathway gene. *ABI3*, *MYB1*, *AREB*: ABA signaling pathways gene. Data are the means of five replicates at 12 days with standard errors shown by vertical bars. Differences among treatments were analyzed by the one-way ANOVA comparison test (*P* < 0.05). Different letters indicate significant differences among treatments. CK, the plant grown at optimal temperature (28°C/18°C); LHT, the plant grown at low night temperature (28°C/6°C); LHT + DA-6, the plant pretreated with DA-6 and grown at LHT.

Compared with other hormones, the ABA content was maintained at a high level in each treatment (Figure 7). The ABA synthesis genes *AAO*, *ABA2*, *NCED4*, and *ZEP1* were significantly upregulated, and *NCED1* was significantly downregulated under LNT. LNT + DA-6 treatment resulted in a decrease in the expression of *AAO*, *NCED1*, *ZEP1*, and an increase in *ABA2* and *NCED1* (Figure 9). Furthermore, LNT treatment induced downregulation of the ABA decomposition gene *CYP707A2*, while *CYP707A1* and *CYP707A2* were upregulated in treated plants with LNT + DA-6 (Figure 9). Gene expression levels of the ABA signal transduction pathway indicate that LNT treatment induced a significant downregulation of *ABI3* and *MYB1* and a significant upregulation of *AREB*. LNT-DA-6 treatment led to an increase in the expression of *ABI3*, *MYB1*, and *AREB* (Figure 9).

**FIGURE 10:**
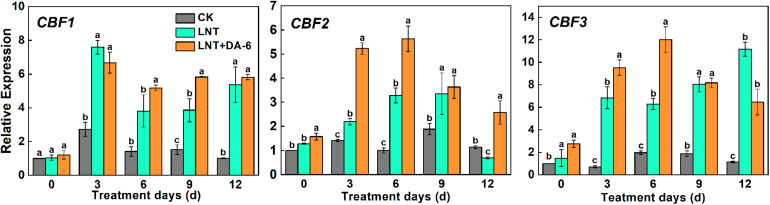
Effect of DA-6 treatment on related gene expression of CBF in tomato leaves under low night temperature. Data are the means of five replicates at 12 days with standard errors shown by vertical bars. Differences among treatments were analyzed by the one-way ANOVA comparison test (*P* < 0.05). Different letters indicate significant differences among treatments. CK, the plant grown at optimal temperature (28°C/18°C); LHT, the plant grown at low night temperature (28°C/6°C); LHT + DA-6, the plant pretreated with DA-6 and grown at LHT.

The expression of the *CBF1*, *CBF2*, and *CBF3* genes was augmented under LNT conditions. Plants treated with LNT+DA-6 showed higher *CBF1* and *CBF2* expression on days 6–12, while *CBF3* increased only in days 0–6 and decreased afterward (Figure 10).

## Discussion

Low temperature is an environmental stress that seriously affects the growth and development of plants (Ding et al., 2020). Plants can adapt to low temperature stress by activating the expression of resistance genes. However, persistent or intense low temperature will damage the plant’s self-regulating system. Chemical elicitors that boost plant defenses are widely regarded as promising tools for plant protection and sustainable agriculture (Wang W. et al., 2020). In recent years, DA-6 has been used as a growth regulator to improve plant resistance and yield. Studies have shown that appropriate DA-6 concentration can enhance root activity, mineral absorption, carbon metabolism, plant height, and stem diameter, and promote plant growth and development (He et al., 2014). Our results show that exogenous also DA-6 improves the adaptability of tomato plants to LNT stress (Figure 1A). Low temperature stress often leads to yellowing of leaves, which is mainly related to the decrease in chlorophyll content in leaves. The synthesis of chlorophyll involves 15 different enzymes encoded by 27 genes. Chlorophyll degradation primarily depends on the action of the enzymes chlorophyllase, Mg-dechelatase, and chlorophyll a oxygenase (Masuda and Fujita, 2008; Hörtensteiner and Kräutler, 2011). Environmental stress not only inhibits chlorophyll synthesis but also promotes chlorophyll degradation (Jin et al., 2019). Previous studies have shown that DA-6 can promote the synthesis of chlorophyll by increasing the conversion efficiency of ALA (aminolevulinic acid) to PBG (Porphobilinogen) and PBG to Uro III (Zhang et al., 2008; Jiang et al., 2012). In this study, exogenous DA-6 significantly increased chlorophyll content in tomato leaves under LNT stress by promoting the expression of chlorophyll synthesis genes and suppressing the expression of chlorophyll degradation genes (Figure 2), which in turn enhanced the photosynthetic efficiency of plants (Figure 1). Consequently, DA-6 regulates the photosynthetic efficiency by raising chlorophyll content in tomato leaves under LNT stress, which ultimately improves the plant resistance to low temperatures.

The PSII is distributed throughout the thylakoid membrane. It is extremely sensitive to temperature and is considered to be the main site of light inhibition (Yan et al., 2013). LNT stress decreases PSII complex activity and PSII receptor side inhibition, which affects electron transfer and D1 protein degradation, eventually causing photodamage of the PSII reaction center (Figures 3A–D) (Murata et al., 2007; Liu et al., 2012; Zhang et al., 2014). LNT + DA-6 treatment resulted in higher Fv/Fm, Y(II), ETR(II), and qP (Figures 3A–D). These results indicate that exogenous DA-6 can prevent excessive accumulation of light energy in the PSII and reduce the photodamage produced by low temperatures.

Plants can dissipate light energy excess in the PSII through NPQ, thereby protecting PSII from photodamage (Lu et al., 2020b). Our observation of higher NPQ after LNT and LNT + DA-6 suggests that although leaves accumulated an excess of energy, energy conversion and other defense regulatory mechanisms (such as heat dissipation) were still in place and allowed plants to consume this excess light energy (Figure 3E). The balance of photodamage and repair maintains PSII activity under stress conditions (Lu et al., 2020b). The PSII protein complex is mainly composed of the PSII light-harvesting complex (LHCII), the OEC, and peripheral antenna proteins (Murata et al., 2007; Nishiyama et al., 2011). The OEC comprises D1, D2, CP43, and CP47 proteins, which are encoded by the *psbA*, *psbD*, *psbB*, and *psbC* genes, respectively (Wittenberg et al., 2014). In this study, LNT stress resulted in downregulation of psbA, psbB, and psbP expression levels, which destroy the stability of the PSII complex and reduce the ability of PSII to absorb light energy and release oxygen. The exogenous DA-6 treatment induced expression of *psbA*, *psbD*, *psbB*, and *psbC* at LNT (Figure 4). These results further indicate that exogenous DA-6 can maintain photochemical activity by keeping PSII stable under LNT stress.

Previous studies have found that under low temperature and low light conditions, PSI is more prone to photoinhibition than PSII, and the repair and/or resynthesis of PSI complexes is very slow compared with the rapid and efficient repair of PSII. Thus, PSI damage is considered to be almost irreversible (Zhang et al., 2011; Huang et al., 2017). LNT treatment led to a decrease in PSI activity and an increase in Y(ND) (Figure 3). The decrease in PSII and PSI activity results in the accumulation of excess light energy in the photosystem and induces a large accumulation of ROS (Figure 6). Exogenous DA-6 promoted PSI activity, upregulated PSI-related gene expression, and led to a significant decrease in Y(ND) and ROS content. This indicates that the application of exogenous DA-6 reduces the oxidative damage in tomato leaves under LNT stress by maintaining photosystem activity.

Chloroplasts are made of a series of membranes, the thylakoid, and stroma. There are also starch grains and osmiophilic granules formed via membrane decomposition in chloroplasts. Normal chloroplasts have a complete membrane system and a large number of closely arranged grana and stroma lamella that provide sufficient space for photosynthesis (Peng et al., 2015). Under low temperature stress, chloroplasts swell, the grana lamella becomes thinner and lower in number. Moreover, the transparency of the envelope and plasma membrane is reduced. Intense and extended low temperature stress can cause the chloroplast stroma to darken, the grana to fall off, the thylakoid membrane and chloroplast membrane to disintegrate, vesicles to accumulate, and overall chloroplast degradation (Figure 5, Karim et al., 2014). Exogenous DA-6 increases photochemical activity under LNT stress because it can inhibit chloroplast degradation (Figure 5) and maintain its structural integrity, stabilize the thylakoid membrane protein, and improve the rate of photosynthetic electron transfer.

Most plant hormones occur as small signaling molecules that regulate plant growth and development, flowering, senescence, and death. Cytokinins were initially discovered as regulators of cell division and are involved in multiple aspects of plant growth and development (Cortleven et al., 2019). More recently, diverse functions for CTK in response to abiotic and biotic stress have been reported (Bielach et al., 2017). Exogenous addition of CTK may increase freezing tolerance in plants and stabilize cell membranes (Jeon et al., 2010; Jiang et al., 2013). ZR is the earliest identified CTK. In this study, LNT treatment resulted in an increase in ZR content. Exogenous DA-6 pretreatment induced a further increase in ZR content at LNT (Figure 7). Previous studies have shown that CTK can prevent chlorophyll and protein degradation, promote chloroplast development, maintain cell viability, and delay plant senescence (Van Staden, 1988; Doležal et al., 2006; Okazaki et al., 2009; Honig et al., 2018). The role of DA-6 in maintaining chlorophyll content and chloroplast structure may be related to CTK accumulation. CTK synthesis starts with the formation of CTK nucleotides, which is catalyzed by isopentenyl transferases (IPTs) (Cortleven et al., 2019). CTK breakdown is catalyzed by CTK oxidase/dehydrogenase (CKX) enzymes (Frebort et al., 2011). Exogenous DA-6 pretreatment increased gene expression related to CTK synthesis and reduced the expression of decomposition genes under LNT (Figure 8). Therefore, DA-6 can improve cold resistance in tomato plants by promoting CTK synthesis.

Abscisic acid content in plants treated with LNT and LNT + DA-6 was higher than in plants treated with CK (Figure 7). ABA regulates not only the growth and development of plants but also the responses to biotic and abiotic stresses (Ma et al., 2018). It is the most important stress signal hormone, and can mediate the signal transduction pathway of cold stress and increase tolerance at low temperatures (Ding et al., 2020). High ABA content at LNT improves the plant’s adaptability to low temperature stress. Exogenous DA-6 may enhance the defense response to LNT by regulating the transcription level of ABA metabolism and signaling pathway genes (Figure 9).

Some studies reported the role of ABA in signaling, although others indicated that its absence or a small role of this hormone and ABA increase is not enough to induce all genes related to cold tolerance. Because of this controversy, many authors suggested that there are two pathways: one that is dependent on ABA and another independent, which might result in the expression of CBF (C-repeat-binding factor) genes (Nievola et al., 2017). Three *CBF/DREB1* genes in *Arabidopsis* play central, redundant roles in cold acclimation (Zhu, 2016; Zhao et al., 2016). *CBF* genes are rapidly and highly induced by low temperatures, and their encoded proteins activate the expression of *COR* genes. This leads to the accumulation of compatible osmolytes and cryoprotective proteins that facilitate cold acclimation and freezing tolerance (Shi et al., 2018; Ding et al., 2020). The induction of *CBF* genes occurred within 30 min after transferring *A. thaliana* plants to 4°C (Thomashow, 2010). In cotton and ryegrass, *CBFs* can also quickly respond to low temperature and reach a peak in 2–4 h (Shan et al., 2007; Tamura and Yamada, 2007). Here, LNT + DA-6 treatment in tomato plants quickly induced the expression of *CBF* genes (Figure 10), indicating that exogenous DA-6 can activate the *CBF* signaling pathway and the expression of COR genes to improve the cold resistance of plants.

## Conclusion

**FIGURE 11:**
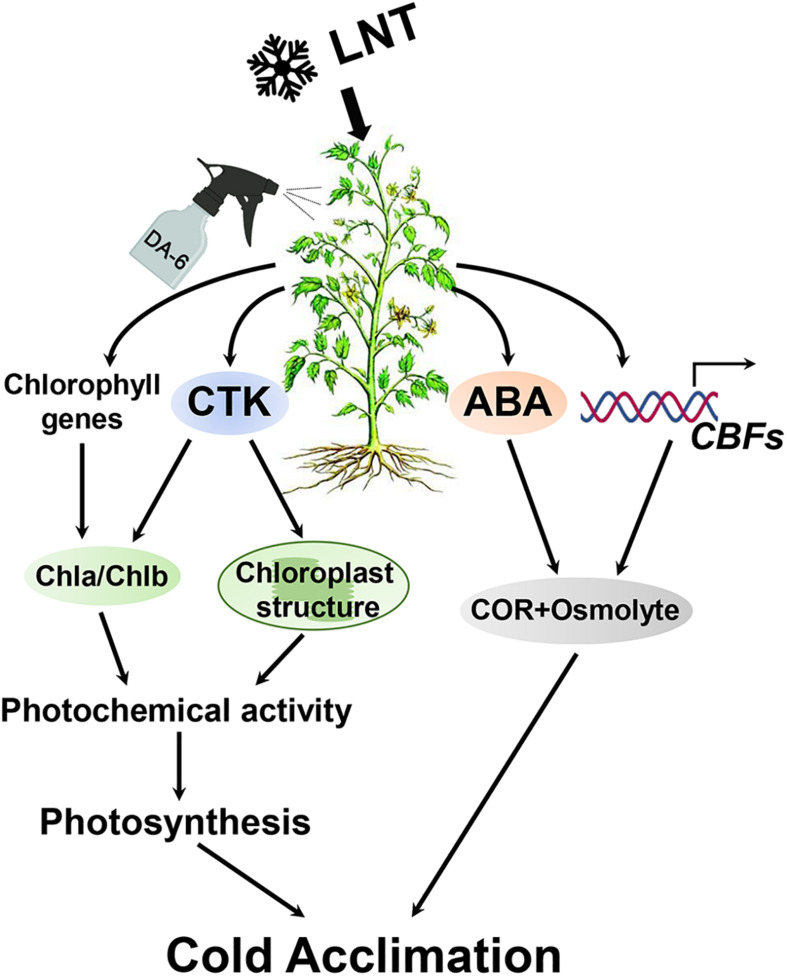
Schematic presentation of DA-6 regulation systematic in tomato leaves under low night temperature stress. Exogenous DA-6 increases the chlorophyll content and stabilizes the chloroplast structure by regulating the expression changes of chlorophyll synthesis and degradation genes and promoting the accumulation of CTK, thereby maintaining the photosynthetic rate. In addition, DA-6 maintains a high level of ABA content under low night temperature stress, and significantly induced CBFs expression, which contribute to the activation of the expression of downstream low temperature response genes and accumulation compatible osmolytes. These will increase the cold acclimation of tomato leaves under low night temperature stress. LNT, low night temperature; DA-6, diethyl aminoethyl hexanoate; CTK, cytokinin; ABA, abscisic acid; CBF, C-repeat-binding factors; COR, cold-regulated genes.

The application of exogenous DA-6 significantly increases the resistance of tomato leaves to LNT and their tolerance to cold stress. Two metabolic pathways are involved in this response: (1) Exogenous DA-6 stabilizes chloroplast structure and increases chlorophyll content by promoting the accumulation of CTK, avoiding severe oxidative damage, and decreasing the photosynthetic rate. (2) Exogenous DA-6 maintains a high level of ABA content and induces *CBF* expression, which activates the expression of cold stress acclimation genes and the accumulation of compatible osmolytes (Figure 11).

## Data Availability Statement

The original contributions presented in the study are included in the article/Supplementary Material, further inquiries can be directed to the corresponding author/s.

## Author Contributions

YL and TL conceived and designed the experiment. PG conducted the experiment. PG, FW, and MQ analyzed the data. JL and PG wrote the manuscript. JL, PG, JG, XY, and YL revised the manuscript. All the authors read and approved the final manuscript.

## Conflict of Interest

The authors declare that the research was conducted in the absence of any commercial or financial relationships that could be construed as a potential conflict of interest.

## Supplementary Material

Supplementary Table 1 Response gene accession numbers and primer sequences described in this study.
