# Supplementary material for: Exogenous DA-6 Improves the Low Night Temperature Tolerance of Tomato Through Regulating Cytokinin
Source: Front Plant Sci. 2021 Feb 4;11:599111. doi: 10.3389/fpls.2020.599111 (PMC7889814; doi:10.3389/fpls.2020.599111)
Supplement: Supplementary Table 1 — Response gene accession numbers and primer sequences described in this study. [file Table_2.DOCX]

**Supplementary Table 1** Response gene accession numbers and primer sequences described in this study

| Gene/protein | Accession No | Primer sequences （5’-3’） |
| --- | --- | --- |
| *HEMA1* | LOC101266935 | 1. AGTGTTGGAAGTTTAAGGCTTTT   R-GCCCTCAGTTTCTTGATGGT |
| *HEMB* | LOC101246744 | F-TTGACTGATGAGGAGTGTGAGG  R-GATATACAAGATTTGCAGGGCTT |
| *HEMC* | LOC101257684 | F-ATCTTACCGTGCAACCTTCC  R-CCATTGTCTCGTCATCACTTCT |
| *HEMD* | LOC101262008 | F-CCGAAGAATGGAAATGACAAG  R-GCTACAGTAACAACGGGAACAGA |
| *HEME1* | LOC101260578 | F-CAAATGGCTATGGCTTTTTCT  R-GGCGACTCACCCGTTCTCT |
| *HEME2* | LOC101250511 | F-ATCGTCTTCTTCTTCTCGCTCT  R-GTACCTCCCTGCTTGTCTCATA |
| *HEMF* | LOC101260673 | F-GAAGCACAAGTAAAAAATCAACAC  R-TTCCAAAGCAGCACAAACAC |
| *HEMG1* | LOC101259974 | F-TCAACCATCCTAGCATTTTCAC  R-CCTTCTTCCCATAAGTATCCATCT |
| *HEMG2* | LOC101251358 | F-ATTTTTTCTGCCTCTCCACAC  R-ATTTCCTCTCTTAGCAATCTTCAT |
| *CHLH* | LOC101244176 | F-AAAATCACCCAAAAAGTTCCAA  R-CTCCTCAACCAAATACCCCAC |
| *CHLD* | LOC101248868 | F-TGTCACAGAAGATAGATTGATTGG  R-TAGTAGTGGTTTGCATGGATGG |
| *CHLM* | LOC101267302 | F-TAAACCCTAATCCCCAACTCA  R-TATCACCGCCTCCGACCT |
| *CHLG* | LOC101246752 | F-ATCAAAGGAGCCAAGCAAGA  R-GAAATGGACCAGACATCAACATAC |
| *PROA* | LOC101248079 | F-GTTGATTGATGATTTGAAGCAGT  R-GGAGCCTAAACAAGGGGATA |
| *PROB* | NM_001310041.1 | F-GTCTACCTCAGGACAGAAGAAAAC  R-TGCCCACACTAAGCTCAAATC |
| *CAO2* | LOC101261422 | F-ATCCTCCTACAGCCACTCTTCC  R-GCTCTGCCCCTCCAATTT |
| *CRD1* | LOC101257518 | F-GTCTCGCTTCTTCTGCCTTT  R-CTCCACCATCCTGTCCAACT |
| *DVR* | LOC101247061 | F-GATGTTGTTGTGTCTTGTCTTGC  R-ATCTTGTTCGCTTATCGGCT |
| *NYC1* | LOC101258872 | F-TGGTTGGTATGGCTTGTGAC  R-TGATTTGGATGGCTTCTTTAGT |
| *HCAR* | LOC101248918 | F-GCATGTGCTTTCTTGGGG  R-AGATTAGGAGACAGTGTCGGCT |
| Gene/protein | Accession No | Primer sequences （5’-3’） |
| *PAO* | LOC543677 | F-TTTGTCGCACCTTGTTACTCA  R-ATATCCCCGTCATACACCTTATT |
| *HO* | NM_001321088.1 | F-TTGCCATTTTTACAACACCTACT  R-ATGACTTCTCTGTCTCTTCCAGAC |
| *PIF4* | LOC101252303 | F-ATGGGAATGGGGATGGGT  R-GCTGAGTTTGCTGTGCTGTATG |
| *EEL* | LOC104649075 | F-GTCGCCACTAAGTCCCGTGT  R-GCTTTCATTTTCGCTTCATCA |
| *FC1* | LOC101248153 | F-CCTGCTTGCCATTCCTCA  R-TCCTCCACCTACACTGCTTCT |
| *FC2* | LOC101247019 | F-CTTCATCTTTCGCTGCTTCTT  R-GCCTTCTTTGCTCTTGGGT |
| *PsbA* | ABC5628 | F-GTATTCGGCGGCTCCCTATT  R-AGATTCATTTTCTGTGGTTTCCC |
| *PsbB* | Solyc01g007500.2 | F-TAACAGGCTTGTATGGTCCC  R-CCTTTGTATAGACGTTGTGGC |
| *PsbC* | Solyc10g047410 | F-TTGGAGGAGAAGGGTGGATT  R-GCAATGAAACCAAAGACGGC |
| *PsbD* | Solyc09g055950.1 | F-GGAGATTTTACTCGTTGGTGTC  R-GTGCAAAGAACCAACCAGAC |
| *PsbP* | Solyc11g008480 | F-CAACAGTGGGAGGAAAAGAG  R-GCAACTCATCTCAGCACCAT |
| *PsaA* | ABC56300 | F-GCACTAGGCCCAATGTGAGT  R-TTTCGAGGAATGGGCCAGAC |
| *PsaB* | ABC56299 | F-AGAGGGGAGTACGTTCGGTG  R-AACTTTGCGTTTGTGGGTGG |
| *PsaC* | Solyc02g011760.1.1 | F-ACCTTGGGACGGTTGTAAGG  R-TCTGTTGGACAGGCGGATT |
| *PsaD* | Solyc06g054260.1 | F-TCAGTAACCCGACCTATCCG  R-CACCACCTGTTGGCATCTC |
| *PsaL* | Solyc06g082940.2 | F-TATCCAACTTGCCTGCCTAC  R-AATGTCAATGCTGGTGCTGT |
| *IPT3* | NM_001279341.2 | F-GTGAATAGCGATAAAATGCAAAT  R-AGAAAGCAACACTCATACCTGG |
| *IPT4* | NM_001257986.1 | F-ACTCTCTATCGACTTAGCCACTCA  R-GGAAACAAAAATCGTACCTTGTACT |
| *IPT5* | XM_010314888.2 | F-TGATTTTTGCTTCTTATGCGTG  R-CTACAAGGTTTTTGATGTTCCAG |
| *CKX2* | NM_001257980.1 | F-CAGGTTTACTGAATAACTGGAGAAC  R-CTAGAAGATTGAGCCATGGATG |
| *CKX3* | NM_001320339.1 | F-TCCATTAACGGCCAAGCTAT  R-TGACCAAGTCCACCAAGAACA |
|  |  |  |
| Gene/protein | Accession No | Primer sequences （5’-3’） |
| *CKX5* | NM_001257978.1 | F-GCCTTTTGTTGATGTCTCTGC  R-GGTCCATGTTTGAATGCTTG |
| *CKX7* | NM_001257979.1 | F-TGGCAATAGATGTCATTTCCTT  R-TCTGCGGTCCGTGCTTAA |
| *AAO* | NM_001247527.1 | F- GTGAGTTTGGTTTTGGCAGTT  R- GCATGGAAACCGGCAAACCT |
| *ABA2* | LOC101254591 | F- ACTTGCCTCCTGAACAAAGG  R- CTTAGCAACATCCTGAGCCA |
| *NCED1* | NM_001247526.2 | F-AGGCAACAGTGAAACTTCCATCAAG  R-TCCATTAAAGAGGATATTACCGGGGAC |
| *NCED4* | LOC101250535 | F-TCGACCACGAAACTAACGAG  R-GCGAAGTCGTGAAGAAATGA |
| *ZEP1* | NM_001309304.1 | F- TCGGACTTGGTCCACTATCA  R- TAACATCAGAGGCATTCCCA |
| *CYP707A1* | NM_001247588.2 | F- ATCACAACCCAGAGTTCTTTCCT  R- CAGATCCTACCACTTCCCACC |
| *CYP707A2* | NM_001375915.1 | F- AGTACAGGTGGTCTATGGTGGG  R- GTTGATGATGTTTTGAGAGAGAGTTT |
| *ABI3* | XM_026031313.1 | F- TTGATACGAGGGGTAAAAGTGAG  R- AACAGCTTGTGCAAATGGAGAT |
| *MYB1* | LOC101261736 | F- GAATTTTCCATCATTGAGGGTTT  R- TCACTTGAGTAGTTTCCAGGGTT |
| *ABRE* | XM_026030430.1 | F- TCCAGCACTCAACTCT  R- TGGCTCCTAAACCTAC |
| *CBF1* | NM_001247194.2 | F- GAGTCGGAAGAAGTTTCAGG  R- TGTAGGCATCAGTTTCCAC |
| *CBF2* | Solyc03g124110.2.1 | F- TTCGATCGGAAGAAGTTTCA  R- CAAGTAATCCTGGCATGGAA |
| *CBF3* | Solyc03g026270.1.1 | F- TGCCGGGTTTACTTACGAAT  R- TCAGCTTCCACATGATCTCC |
| *Actin* | Solyc11g005330 | F-TGTCCCTATTTACGAGGGTTATGC  R-CAGTTAAATCACGACCAGCAAGAT |
